# Supplementary material for: Dynalign II: common secondary structure prediction for RNA homologs with domain insertions
Source: Nucleic Acids Res. 2014 Nov 21;42(22):13939–48. doi: 10.1093/nar/gku1172 (PMC4267632; doi:10.1093/nar/gku1172)
Supplement: SUPPLEMENTARY DATA [file supp_gku1172_nar-02021-z-2014-File012.zip › manual/Text/AllSub.html]

RNAstructure Command Line Help: AllSub


|  |  |  |
| --- | --- | --- |
|  | RNAstructure Command Line Help AllSub | - Contents - Index |
| AllSub is used to generate all possible secondary structures within a small energy increment above the lowest free energy structure. USAGE: AllSub <seq file> <ct file> [options] Required parameters: <seq file> \| The name of a sequence file containing input data. Note that lowercase nucleotides are forced single-stranded in structure prediction.; <ct file> \| The name of a CT file to which output will be written. Options which don't require added values: -d, -D, --DNA \| Specify that the sequence is DNA, and DNA parameters are to be used. Default is to use RNA parameters.; -h, -H, --help \| Display the usage details message. Options which require added values: -a, -A --absolute \| Specify a maximum absolute energy difference in kcal/mol. All structures between the lowest free energy and the lowest free energy plus this parameter will be generated. Note that suboptimal structures are generated until either the absolute energy difference is reached or until the maximum percent energy difference is reached (below). Default is determined by the length of the sequence.; -c, -C, --constraint \| Specify a folding constraints file to be applied. For AllSub, current supported constraints are force pairs, force a nucleotide to be single stranded, and force a nucleotide to be double stranded. Default is to have no constraints applied.; -p, -P, --percent \| Specify a maximum percent energy difference in folding free energy change for generating suboptimal structures. All suboptimal structures are generated at two separate energy thresholds. The first is the lowest folding free energy. The second is lowest folding free energy minus a difference fraction. This difference fraction is calculated by dividing the percent difference by 100, multiplying that value by the lowest folding free energy, and subtracting the resultant value from the lowest folding free energy. Note that suboptimal structures are generated until either the maximum percent energy difference is reached or until the absolute energy difference is reached (above). Default is determined by the length of the sequence.; -t, -T, --temperature \| Specify the temperature at which calculation takes place in Kelvin. Default is 310.15 K, which is 37 degrees C. References: 1. Reuter, J.S. and Mathews, D.H. "RNAstructure: software for RNA secondary structure prediction and analysis." *BMC Bioinformatics*, 11:129. (2010). 2. Duan, S., Mathews, D.H. and Turner, D.H. "Interpreting oligonucleotide microarray data to determine RNA secondary structure: application to the 3' end of Bombyx mori R2 RNA." *Biochemistry*, 45:9819-9832. (2006). 3. Wuchty, S., Fontana, W., Hofacker, I.L. and Schuster, P. "Complete suboptimal folding of RNA and the stability of secondary structures." *Biopolymers*, 49:145-165. (1999). | | |
| Visit The Mathews Lab RNAstructure Page for updates and latest information. | | |
